# Establishment of a Refined Oral Glucose Tolerance Test in Pigs, and Assessment of Insulin, Glucagon and Glucagon-Like Peptide-1 Responses

**DOI:** 10.1371/journal.pone.0148896

**Published:** 2016-02-09

**Authors:** Elin Manell, Patricia Hedenqvist, Anna Svensson, Marianne Jensen-Waern

**Affiliations:** Department of Clinical Sciences, Faculty of Veterinary Medicine and Animal Science, Swedish University of Agricultural Sciences, Uppsala, Sweden; Van Andel Research Institute, UNITED STATES

## Abstract

Diabetes mellitus is increasing worldwide and reliable animal models are important for progression of the research field. The pig is a commonly used large animal model in diabetes research and the present study aimed to refine a model for oral glucose tolerance test (OGTT) in young growing pigs, as well as describing intravenous glucose tolerance test (IVGTT) in the same age group. The refined porcine OGTT will reflect that used in children and adolescents. Eighteen pigs were obtained one week after weaning and trained for two weeks to bottle-feed glucose solution, mimicking the human OGTT. The pigs subsequently underwent OGTT (1.75 g/kg BW) and IVGTT (0.5 g/kg BW). Blood samples were collected from indwelling vein catheters for measurements of glucose and the diabetes related hormones insulin, glucagon and active glucagon-like peptide-1. The study confirmed that pigs can be trained to bottle-feed glucose dissolved in water and thereby undergo an OGTT more similar to the human standard OGTT than previously described methods in pigs. With the refined method for OGTT, oral intake only consists of glucose and water, which is an advantage over previously described methods in pigs where glucose is given together with feed which will affect glucose absorption. Patterns of hormonal secretion in response to oral and intravenous glucose were similar to those in humans; however, the pigs were more glucose tolerant with lower insulin levels than humans. In translational medicine, this refined OGTT and IVGTT methods provide important tools in diabetes research when pigs are used as models for children and adolescents in diabetes research.

## Introduction

Diabetes mellitus (DM) is increasing worldwide and the Wold Health Organization (WHO) estimates that DM will be the seventh leading cause of death in 2030 [1] and it is alarming that DM is increasing in children and adolescents [2]. Both patients with type 1 diabetes (T1D) and type 2 diabetes (T2D) have a shorter life expectancy compared to the general population [3, 4] and the disease is associated with long-term complications such as cardiovascular disease [4], renal failure [5] and vision impairment [6, 7]. Robust animal models are crucial for progressions in the diabetes research field. The pig is an excellent large animal model since it is anatomically and physiologically very similar to humans [8] and therefore can bridge the gap in translational medicine between rodent models and humans.

Oral glucose tolerance test (OGTT) is widely used in the clinic to diagnose human patients with impaired glucose tolerance and diabetes mellitus based on WHO recommendations [9]. In addition, OGTT is a very valuable tool in diabetes research and is commonly used to evaluate disease progression, effect of treatments and to learn more about physiological and pathophysiological conditions. However, OGTT in pigs is cumbersome. The most commonly used method for glucose tolerance testing in pigs is to feed glucose (2 g/kg) along with a small amount of feed [10]. This mixed meal approach may be complicated by incomplete consumption of the glucose-containing meal, and the diet itself may alter the glucose absorption. For example, fibres affect postprandial absorption of glucose giving rise to attenuated blood glucose and insulin concentrations [11, 12], and decelerate gastric emptying [12, 13]. The use of different amounts and content of feed in previous studies have resulted in variations in the glucose tolerance curves [14,16]. In translational medicine where pigs are used as models for humans, there is a need to refine the porcine OGTT model to make it more standardised and closer to the human standard OGTT.

Another glucose tolerance test commonly used in research is the intravenous glucose tolerance test (IVGTT), which is more easy to test in different species as it does not require oral intake of glucose. However, IVGTT cannot always replace OGTT. Oral ingestion of glucose elicits a greater insulin release from the pancreatic beta-cells than intravenous (i.v.) administration of glucose [17] due to the release of the incretin hormones glucagon-like peptide-1 (GLP-1) and gastric inhibitory polypeptide (GIP) [18, 19]. OGTT is needed to study the incretin effect, which is impaired in both T1D [20] and T2D [21, 22]. GLP-1 analogues are widely used as medication for patients with T2D, and have recently also been suggested for treatment of T1D [23, 24]. Despite many years of research, there are still gaps in the knowledge of the physiological effects of endogenous and exogenous GLP-1.

The present study aimed to 1) refine and standardise the procine OGTT model to make it comparable to human OGTT, 2) to describe IVGTT in the same individuals, and 3) to investigate the hormonal responses to oral and intravenous glucose loads with respect to insulin, glucagon and GLP-1 in growing pigs as a model for human children and adolescents.

## Materials and Methods

All procedures were approved by the Ethics Committee for Animal Experimentation, Uppsala, Sweden (Permit number C99/14); and the Guide for the care and use of laboratory animals of the National Research Centre was followed.

### Animals

Two litters of high-health pigs were obtained from the University herd (Swedish Livestock Research Centre, Lövsta, Sweden). The first litter (L1; Yorkshire x Swedish Landrace; females n = 4, males n = 4) was used to establish a way of voluntarily oral intake of glucose dissolved in water. The second litter (L2; Yorkshire x Swedish Landrace x Hampshire; females n = 5, males n = 5) was used to assess hormonal responses to oral and i.v. glucose. The pigs were acquired one week after weaning, and after a two week acclimatisation period the mean weights were 17.1 ± 0.4 kg for L1 and 32.7 ± 0.8 kg for L2. The animals were housed at the Department of Clinical Sciences in individual pens measuring 3 m^2^, within sight and sound of one another. Straw and wood shavings were used as bedding. A 14:10 h light/dark schedule (lights on at 06:00 h) was applied and an infrared lamp (24 h) was provided in each pen. The room temperature was 16–18°C. The pigs were fed commercial finisher diet without growth promoters and antimicrobals (SOLO 330, Lantmännen, Sweden) twice daily (07:00 and 15:00), the amount depending on BW according to the Swedish University of Agricultural Sciences regimen for growing pigs [25]. Water was provided *ad libitum*.

### Acclimatisation and Training

**Fig 1:**
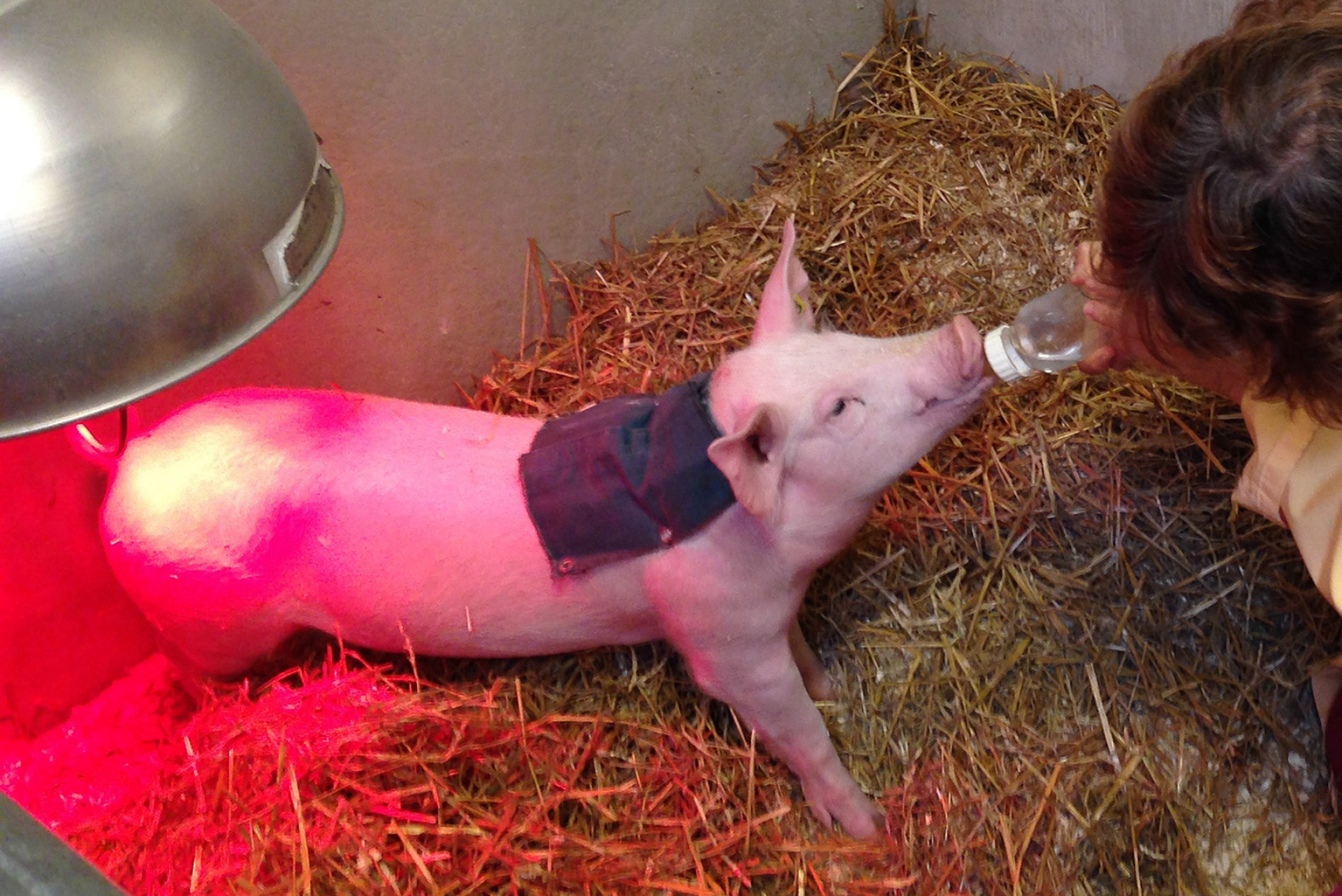
Bottle-feeding training of an eight week old pig.

During a two week acclimatisation period the pigs were handled frequently and trained to step onto an electronic scale. This period was used to accustom the pigs to the staff to ensure that any stress during experiments was avoided and to see that the pigs were healthy at admission to the experiments. During the acclimatisation period, the pigs were also trained to bottle-feed glucose dissolved in water (Fig 1). With L1 bottle-feeding was initiated on day 10 of the acclimatisation period and the pigs were then bottle-fed Mondays to Fridays except on days of IVGTT. With L2 bottle-feeding was initiated on the day of arrival and carried out 7 days a week, the more frequent bottle feeding in L2 was carried out since a decline in bottle-feeding ability was observed over time in L1.

### Surgical Implantation of Indwelling Vein Catheters

After acclimatisation, an indwelling silicone catheter (SIL-C70 with rounded tip; Instec Solomon, Plymouth Meeting, PA, USA) was inserted into *vena jugularis dexter* under aseptic conditions and general anaesthesia as previously described [48]. After anaesthesia induction, blood samples were collected in EDTA- and serum-tubes. EDTA-preserved blood was analysed for total and differential white blood cells (WBC) and Hb by an electronic cell counter validated for porcine blood (Advia 2120, Siemens, Erlangen, Germany). Serum samples were analysed for creatinine (CREA) and enzyme activities of aspartate aminotransferase (ASAT), alanine aminotransferase (ALAT), ɣ-glutamyltransferase (GT) and glutamate dehydrogenase (GLDH) by automated equipment (Architect C4000, Abbott Diagnostics, North Ryde, Australia). During anaesthesia, cardiovascular and respiratory parameters were monitored. The silicone catheter was tunnelled subcutaneously and exteriorised on the back between the scapulas. The surgical procedure was finished within 45 min. Procaine benzylpenicillin (Penovet^®^ vet, 300 mg/mL; Boehringer Ingelheim Vetmedica, Ingelheim am Rhein, Germany) was given preoperatively at a dose of 20 mg/kg BW intramuscularly and benzylpenicillin (Bensylpenicillin^®^ Meda, 100 mg/mL; Meda, Solna, Sweden) was given postoperatively twice daily for two consecutive days at a dose of 6 mg/kg BW i.v. The catheters facilitated frequent blood sampling during glucose tolerance tests (GTTs). Catheters were flushed twice daily with heparinised saline 100 IE/ml (Heparin LEO, 5000 IE/ml; Leo Pharma, Ballerup, Denmark).

### Oral and Intravenous Glucose Tolerance Tests

**Fig 2:**
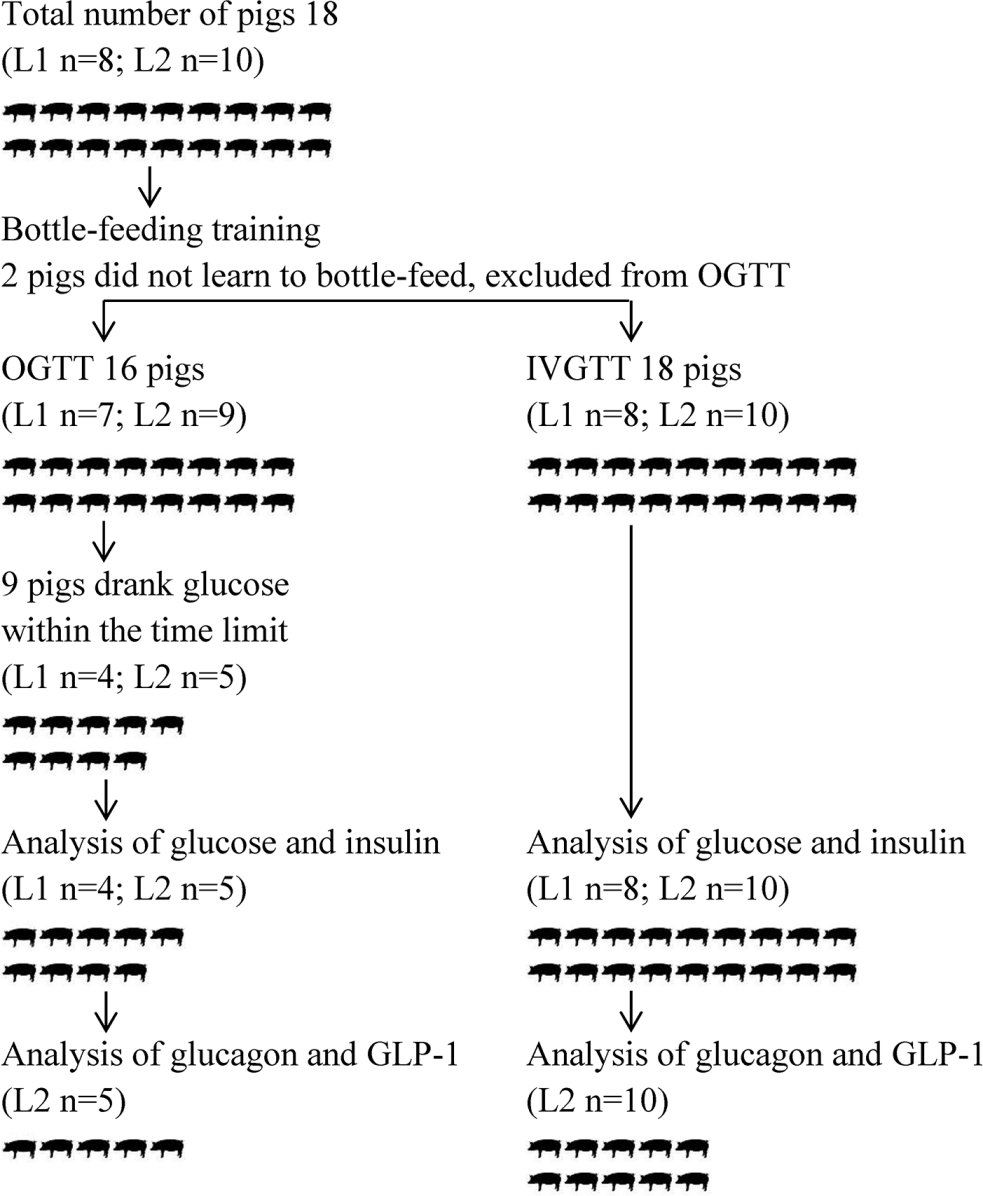
Scheme of the pigs included in IVGTT and OGTT from litter 1 (L1; Yorkshire x Swedish Landrace) and litter 2 (L2; Yorkshire x Swedish Landrace x Hampshire).

Each pig underwent IVGTT (n = 18), and those that were successfully trained to bottle-feed glucose solution also underwent OGTT (n = 16); the pigs were awake during all GTTs. Nine pigs underwent OGTT before IVGTT, and seven pigs underwent OGTT after IVGTT. GTTs were separated by an interval of at least 48 hours. A scheme of the pigs in GTTs is presented in Fig 2. Glucose tolerance was tested after an 18 hour overnight fast. The pens were cleaned from straw and wood shavings and the pigs were provided vetbeds during the fast and GTTs. Water was withdrawn one hour before GTTs. Blood samples were collected before, and 2, 5, 10, 20, 30, 45, 60, 90, 120 and 180 min after glucose intake/infusion. Blood samples from L1 were collected in EDTA tubes for measurement of glucose and insulin concentrations, and from L2 in BD P800 tubes (BD Diagnostics, Franklin Lakes, USA) containing protease-, esterase- and dipeptidyl peptidase IV-inhibitors, and EDTA for measurements of glucose, insulin, glucagon and active GLP-1. Blood glucose was measured immediately, thereafter blood samples were centrifuged, and plasma was stored at -80°C until analysed.

OGTT: The pigs were bottle-fed an oral glucose load (Glukos APL Pulver till oral lösning, APL, Stockholm, Sweden) of 1.75 g/kg BW mixed with 2 ml water/g glucose; the glucose solution had to be consumed within 5 min. Nine pigs drank the glucose solution within the time limit and during OGTT blood samples were collected from these nine pigs.

One pig underwent a second OGTT with an oral glucose load of 2.5 g/kg BW, blood was collected in BD P800 tubes and was used primarily to investigate if this glucose load would give a larger increase in plasma active GLP-1 levels during OGTT, since GLP-1 response to 1.75 g/kg BW was low.

IVGTT: Glucose (Glucose Fresenius Kabi, 500 mg/ml, Fresenius Kabi, Halden, Norway) was infused intravenously at a dose of 0.5 g/kg BW over one minute.

### Blood Analyses

Blood glucose concentrations were measured with test strips (Accu-Chek, Roche Diagnostics, Basel, Switzerland; validated for porcine blood at the Department of Clinical Chemistry, SLU). Plasma insulin concentrations were determined by Porcine Insulin ELISA (10-1200-01, Mercodia, Uppsala, Sweden). All samples were run in duplicates with CV <10%. Inter-assay variations were 7%, 8% and 9% for 0.520, 0.161 and 0.043 μg/L standards, respectively. Results were converted from μg/L to pmol/L using a conversion factor of 174, as recommended by the manufacturer. Plasma glucagon concentrations were measured by Glucagon ELISA (10-1281-01 Mercodia, Uppsala, Sweden). All samples were run in duplicates with CV <10%. Inter-assay variations were 8%, 9% and 9% for 68.4, 24.7 and 8.47 pmol/L standards, respectively. Plasma concentrations of active GLP-1 were analysed by Glucagon-Like Peptide-1 (Active) ELISA (EGLP-35K, Merck Millipore, St Charles, Missouri, USA). This assay measures 100% of both GLP-1(7–36 amide) and GLP-1(7–37), but not the inactive peptides. All samples were run in duplicates with CV <10%. Inter-assay variations were 5% and 7% for 40.0 and 7.5 pmol/L standards, respectively.

### Euthanasia and Post Mortem Examination

At the end of the experiment the pigs were euthanised with an i.v. overdose of pentobarbital sodium (Euthasol vet, 400 mg/ml; Virbac, Reading, Carros, France). The pigs underwent gross examination post mortem.

### Calculations and Statistical Analyses

All values are presented as mean ± SEM unless otherwise stated. Comparisons were made by Friedman repeated measures ANOVA on ranks and the statistical significance of differences was determined by Wilcoxon signed rank test. P values <0.05 were considered to be statistically significant. AUC values were calculated by the trapezoid rule and are presented as the total area. Due to large variations in fasting GLP-1 values (range 3.5–15.9 pmol/L) baseline subtracted GLP-1 concentrations, *id est* change from fasting level, were used. All analyses were run by Sigma Plot 13.0 (Systat Software Inc).

## Results

All pigs became well accustomed to the staff during the acclimatisation period and were very calm during the experiments. None of the pigs showed any clinical signs of disease during acclimatisation and blood concentrations of Hb, total and differential WBC counts and serum levels of ALAT, ASAT, GLDH, GT and CREA were within the reference range for all pigs. One pig was euthanised due to fever and reduced general condition, post mortem examination revealed pneumonia and thrombophlebitis; this pig had already participated in GTTs. For the remaining 17 pigs, post mortem examination showed no signs of disease.

### Bottle-Feeding

While most pigs were very easily trained to drink the glucose solution, some pigs were hesitant. The hesitant pigs were given meat broth for a couple of days, thereafter the glucose content in the bottle was gradually increased and the meat broth content gradually decreased. In this way even the pigs that first were unwilling to drink learned to like the glucose solution. Two out of 18 pigs refused to drink both glucose and meat broth; these two pigs could therefore not undergo OGTT. In L1 (not bottle-fed on weekends) the sucking from the feeding-bottle declined somewhat over time and the pigs started to bite more on the rubber teats. In L2 (bottle-fed every day) the pigs continued to suck from the feeding-bottle and improved their sucking behaviour throughout the study.

### Glucose Tolerance Tests

**Fig 3:**
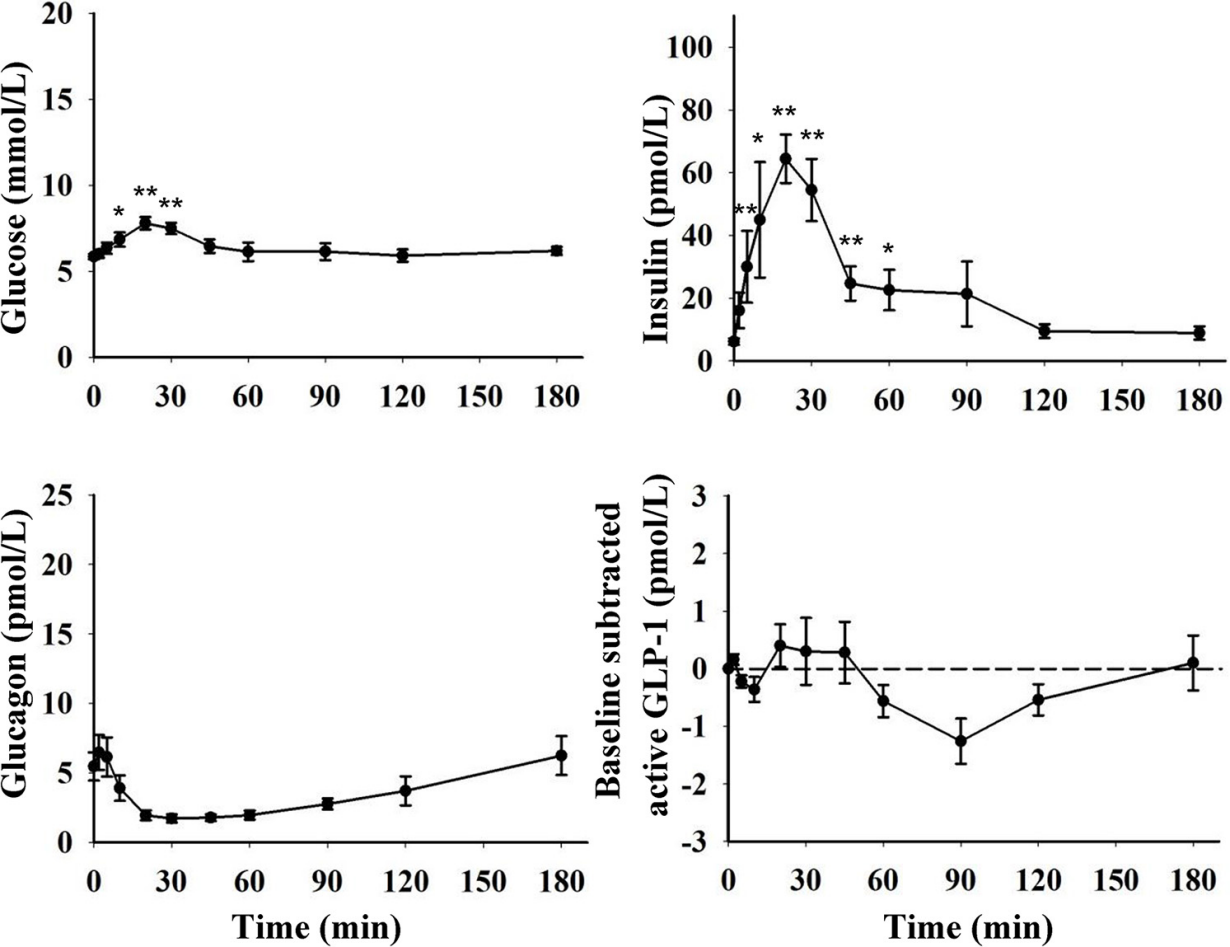
Blood glucose concentrations and plasma levels of insulin, glucagon and baseline subtracted (change from fasting levels) active GLP-1 during OGTT (1.75 g/kg BW, n = 9 for blood glucose and plasma insulin, n = 5 for plasma glucagon and active GLP-1) in growing pigs. * indicate a significant increase and † a significant decrease compared to fasting levels (*, † = p<0.05; **, †† = p<0.01; ***, ††† = p<0.001).

**Fig 4:**
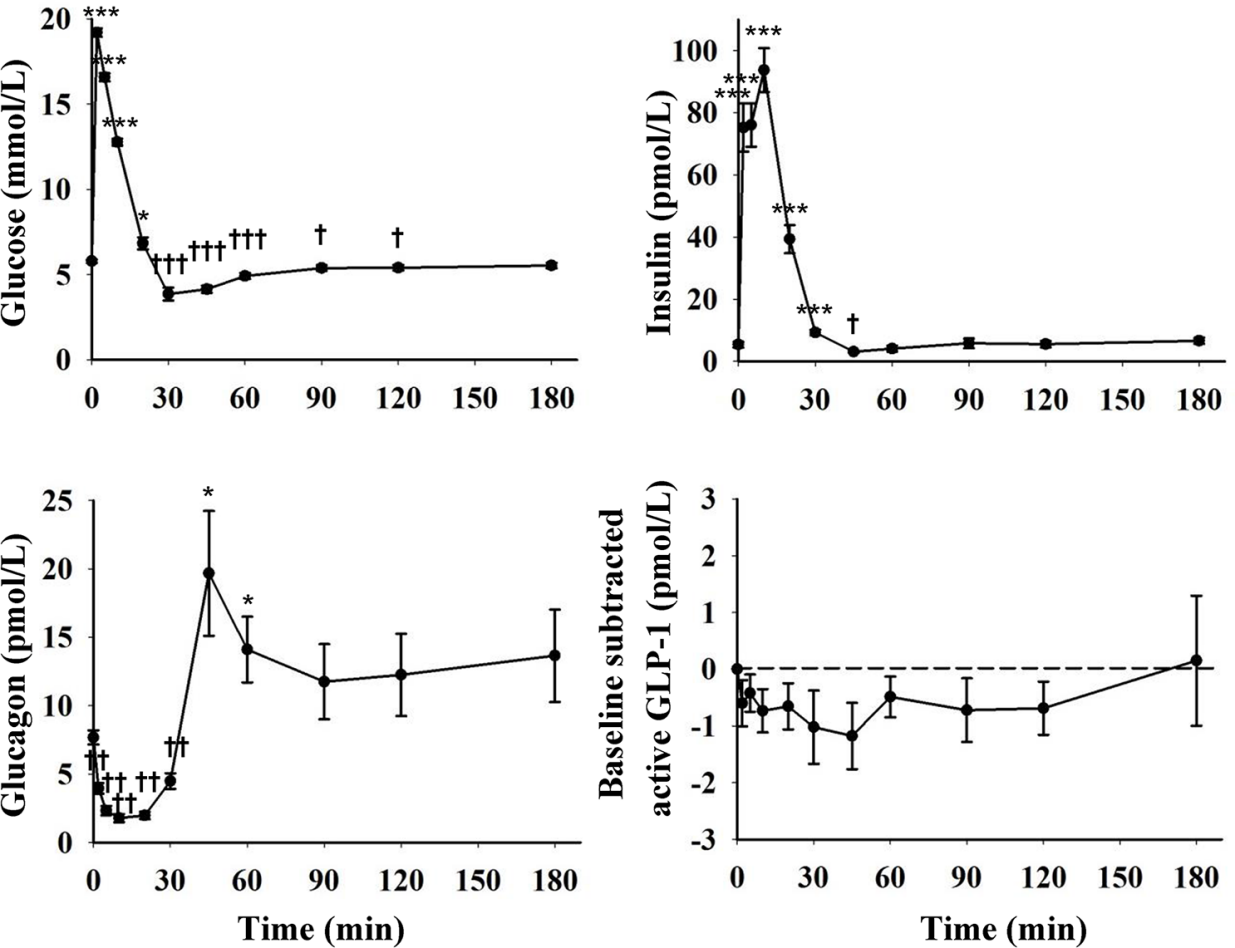
Blood glucose concentrations and plasma levels of insulin, glucagon and baseline subtracted (change from fasting levels) active GLP-1 during IVGTT (0.5 g/kg BW, n = 18) in growing pigs. * indicate a significant increase and † a significant decrease compared to fasting levels (*, † = p<0.05; **, †† = p<0.01; ***, ††† = p<0.001).

Fasting levels of blood glucose and plasma insulin, glucagon and active GLP-1 are presented in Table 1, and levels during GTTs are presented in Fig 3 (OGTT) and Fig 4 (IVGTT).

**Table 1:** Fasting levels of glucose, insulin, glucagon and GLP-1.

**Litter 1**			
	**All pigs (n = 8)**	**Females (n = 4)**	**Males (n = 4)**
**Weight (kg)**	18.5 (17.2–20.1)	18.9 (18–20.1)	18.2 (17.2–19.3)
**Fasting glucose (mmol/L)**	5.8 (5.3–6.7)	5.8 (5.3–6.7)	5.9 (5.4–6.3)
**Fasting insulin (pmol/L)**	5.7 (2.6–12.0)	5.9 (2.6–9.7)	5.5 (3.0–12.0)
**Litter 2**			
	**All pigs (n = 10)**	**Females (n = 5)**	**Males (n = 5)**
**Weight (kg)**	39.3 (35–43)	40.5 (36.5–43)	38.1 (35–40.5)
**Fasting glucose (mmol/L)**	5.7 (5.2–6.3)	5.5 (5.2–6.2)	6.0 (5.4–6.3)
**Fasting insulin (pmol/L)**	5.1 (0.5–11.8)	5.5 (1.7–11.8)	4.7 (0.5–11.3)
**Fasting glucagon (mmol/L)**	7.7 (5.7–10.9)	7.6 (5.7–10.9)	7.7 (5.8–9.0)
**Fasting active GLP-1 (mmol/L)**	8.8 (3.5–15.9)	7.9 (3.5–15.9)	9.7 (6–12.8)

Weight and fasting concentrations of blood glucose and plasma insulin, glucagon and active GLP-1 before IVGTT in litter 1 (Yorkshire x Swedish Landrace, 2 months old) and litter 2 (Yorkshire x Swedish Landrace x Hampshire, 3 months old). Values are mean (range).

**Fig 5:**
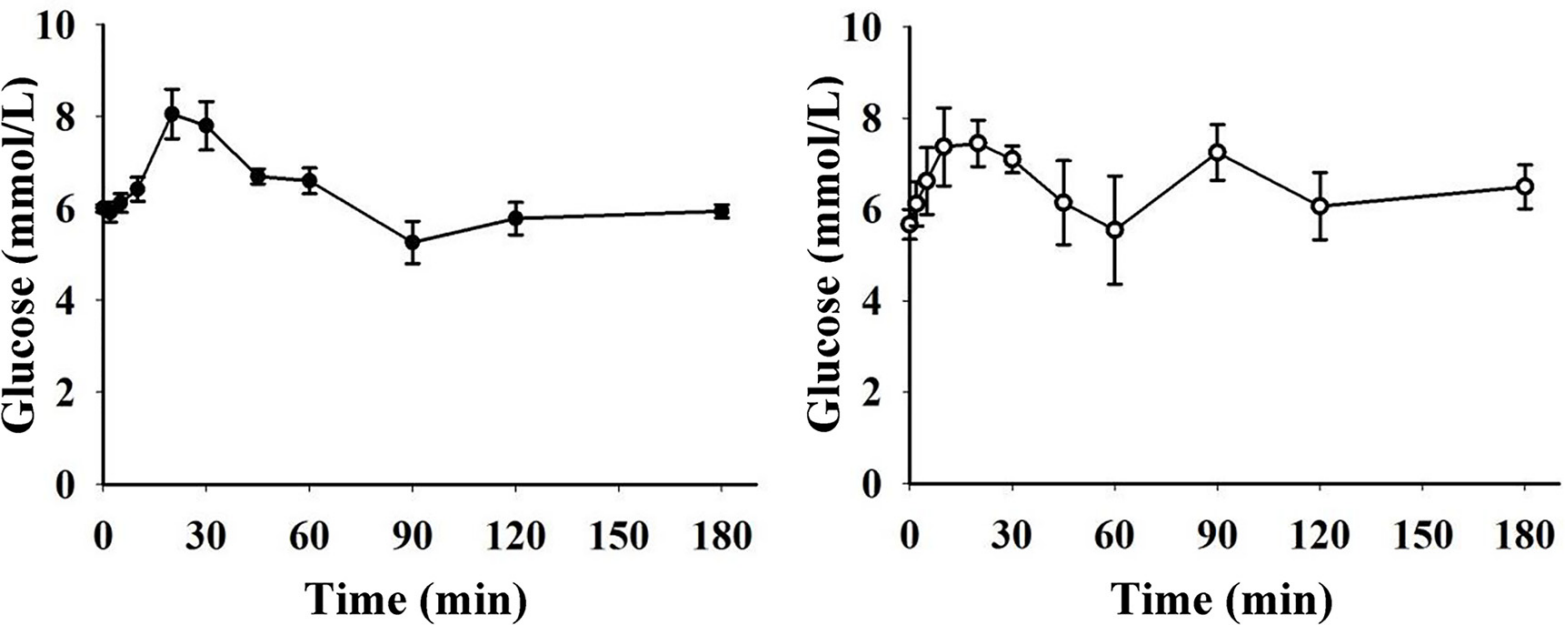
Blood glucose concentrations during OGTT (1.75 g/kg BW) in growing pigs displaying monophasic (n = 5; filled circles) and biphasic (n = 4; open circles) curves.

During OGTT, blood glucose concentrations were increased 10–30 minutes after glucose intake, thereafter levels decreased to values not different from fasting levels at 45 min and onwards. Two patterns of glucose curves during OGTT were identified (Fig 5), monophasic (n = 5) and biphasic (n = 4). In the monophasic curves, peak blood glucose concentrations (range 7.1–9.7 mmol/L) were seen within 30 min after glucose intake, and thereafter the concentrations returned to basal values. In the biphasic curves, two peaks were observed. Blood glucose concentrations increased to a range of 7.1–8.8 mmol/L within 30 min after glucose intake, and returned to basal values before a second peak was seen (range 7.5–8.1 mmol/L) within 60–120 min after glucose intake. All pigs with biphasic curves were females. Monophasic and biphasic curves were observed in both litters.

During OGTT, plasma levels of insulin were gradually increased with peak values within 10–30 min in all pigs. The insulin concentrations gradually decreased and from 90 min onwards no differences from fasting values were observed. For each individual insulin curve, levels increased and decreased in parallel with the corresponding blood glucose curve. In 4 out of the 5 pigs analysed for glucagon, an initial early rise in glucagon levels was observed followed by a gradual decrease. Within 30 minutes after glucose intake, plasma glucagon concentrations were ~70% lower than fasting levels. At the 20–60 min sampling occasions, the decrease was close to reach statistical significance (p = 0.063). Thereafter glucagon levels gradually increased to basal levels. In pigs with biphasic glucose curves, the second blood glucose peak was not preceded by a glucagon peak. After glucose intake GLP-1 levels were increased in 4 out of 5 pigs by 8–39% from baseline, with peak levels (Δ0.9–2.6 pmol/L) within 20–45 min.

**Fig 6:**
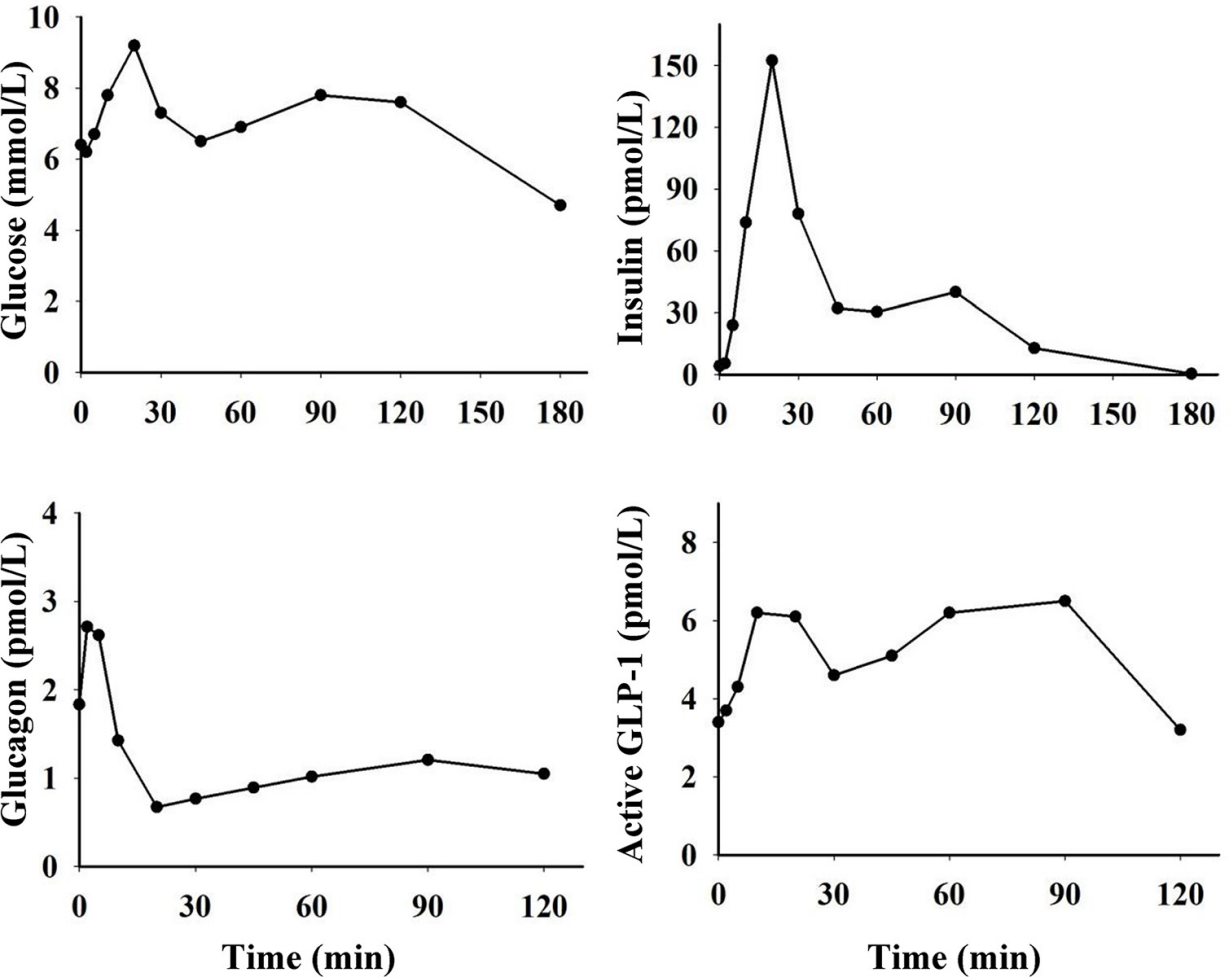
Blood glucose concentrations and plasma levels of insulin, glucagon and active GLP-1 during OGTT (2.5 g/kg BW) in one male 10 week old pig.

Blood glucose concentrations and plasma levels of insulin, glucagon and active GLP-1 for the pig that underwent a second OGTT with an oral glucose load of 2.5 g/kg BW are presented in Fig 6. The AUC of glucose for this pig was 12% higher during 2.5 g/kg OGTT (1260 mmol x min x L^-1^) compared to 1.75 g/kg OGTT (1126 mmol x min x L^-1^). A larger GLP-1 response was observed with this larger glucose load, compared to 1.75 g/kg OGTT. An 80% increase in plasma concentration of active GLP-1 was seen within 10 min after glucose intake. After the peak, GLP-1 levels gradually decreased to a level comparable to the fasting level, and thereafter a second peak (80–90% increase) was observed at 60–90 min after glucose intake.

During IVGTT, peak blood glucose concentrations were observed 2 min after glucose infusion and thereafter gradually decreased to reach values below fasting levels after 30 min. After glucose nadir (3.9 ± 0.4 mmol/L), glucose concentrations were slowly elevated again to reach values comparable to fasting levels at 180 min. Plasma insulin levels increased 14-fold within 2 min and peak concentrations (93.7 ± 7.1 pmol/L) were seen at 10 min after glucose infusion and remained elevated also at 20 and 30 min. Thereafter insulin levels decreased below fasting levels, but had returned to basal levels at 60 min. Plasma glucagon levels were rapidly suppressed after glucose infusion reaching nadir (1.6 ± 0.3 pmol/L) within 5–20 min. Glucagon levels thereafter increased in response to the subsequent hypoglycaemia and was elevated compared to fasting levels at 45 min, but had declined to basal levels at 90 min. Plasma levels of active GLP-1 did not change during IVGTT.

## Discussion

The results from the present study confirm that oral glucose tolerance can be tested in pigs in a similar manner as is done in humans. Pigs are commonly used as a large animal model in research due to its many similarities to humans [8]. Robust and standardised animal models are important to be able to minimise animals used in research, and will result in an improved reliability of research results. The refined porcine OGTT model in young growing pigs, established in the present experiment, provides an important new model for metabolic research, especially for childhood diabetes. Both T1D and T2D are increasing in children and adolescents [2]. T1D has always been a disease where childhood onset is most common; however T2D which has traditionally been recognised as a disease in the adult population is now affecting younger people and has increased fast in children and adolescents during the recent years [2, 26]. Research on physiology, prevention and treatment of DM is therefore of uttermost importance.

The OGTT model presented in the present experiment is a refinement of the mixed meal OGTT used in pigs, and the new model has major advantages compared to the previously described method. In the present study, the effects of glucose alone could be studied as the pigs drank only glucose (dissolved in water) i.e. the results were not affected by any diet, which was the case in other studies [14–16]. In addition, the glucose was contained within the feeding-bottle and incomplete consumption could easily be detected and measured by checking the bottle content after the time limit. A comparison of human standard OGTT, the refined porcine OGTT model and porcine mixed meal OGTT is summarised in Table 2. The key to get the pigs to voluntarily bottle-feed glucose dissolved in water is to train them a lot and to spend time with the animals so that they get accustomed to the staff and the procedures. The pigs improved their bottle-feeding skills the more they trained, and it is recommended to bottle-feed the pigs once daily for at least 2 weeks before the OGTT.

**Table 2:** Comparison of human standard OGTT, refined porcine OGTT model and porcine mixed meal OGTT.

	Human OGTT	Refined porcine OGTT method	Traditional porcine mixed meal OGTT
**Nutritional content**	Glucose + water	Glucose + water	Glucose + feed (content varies between studies)
**Time of glucose intake**	Standardised, within 5 minutes	Standardised, within 5 minutes	Seldom reported, not standardised (?)
**Intestinal glucose absorption**		Similar to human OGTT	Attenuated due to fibre content of meal
**Shape of glucose curves**	Monophasic and biphasic described	Monophasic and biphasic described	Only monophasic described
**Comparison between different studies**	Easy due to standardised test	Easy due to standardised test	Difficult due to the use of different feed in different studies

In the present study, fasting levels of blood glucose and plasma glucagon and active GLP-1 were in the same range as levels measured in humans [27, 28]. Similar to humans [29], there were large inter-individual variations in fasting levels of GLP-1 among the pigs in the present experiment. Even though the pigs were of the same age, lived under the same housing conditions and were fed the exact same meal size and content, fasting levels of active GLP-1 ranged from 3.5 to 15.9 pmol/L. Plasma insulin levels vary with age and stage of puberty in both humans and pigs [16, 30, 31]. In the present experiment, young pre-pubertal pigs were used and their fasting insulin levels were somewhat lower compared to the insulin levels of pre-pubertal children [30–32].

The time from oral glucose intake to peak blood glucose levels during OGTT in the present experiment was similar to OGTT in humans. However, the pigs were more efficient than humans in eliminating glucose from the circulation [21, 33]. When interpreting the individual blood glucose curves two patterns were identified, monophasic and biphasic curves. Biphasic curves have been described in humans with a strong association with the female sex [34]. Interestingly, all pigs with biphasic curves in the present experiment were females. In humans, biphasic glucose curves have been associated with lower BMI and better glucose tolerance, insulin sensitivity and beta cell function [35, 36]. Although the underlying biological mechanisms remain to be elucidated, it might be important to keep this phenomenon in mind. In the present study, one pig showed a second peak with 8.1 mmol/L blood glucose at the 120 min sampling occasion, i.e. above the limit for impaired glucose tolerance [9]. In the clinic, when blood is sampled less frequently and the diagnosis of impaired glucose tolerance is based on 2-h glucose levels, individuals with biphasic curves may be captured on their second peak at 120 min. These individuals might therefore be diagnosed with impaired glucose tolerance although they represent a healthier phenotype.

After an early increase in plasma glucagon levels after oral glucose intake, glucagon was suppressed as insulin levels increased in response to the elevated blood glucose levels. The decrease in glucagon during OGTT was close to reaching statistical significance and will be further evaluated in future studies. The glucagon pattern seen during OGTT was fairly similar to that seen during human OGTT, though the glucagon levels in the pig seem to return to basal levels slightly faster than in humans [15, 37]. Four out of five pigs had an initial early rise in glucagon levels during OGTT with peak values within 10 min, before any change in blood glucose concentration. In the first part of the OGTT, blood was sampled at 2, 5, 10 and 20 min, which is more frequent than in most other OGTT experiments. Without the early frequent sampling, the glucagon peaks would have been missed as glucagon levels lower than in the fasting state were seen in all pigs at 20 min. Hormonal secretion in response to oral glucose is complex. GIP is well known to stimulate beta cells to release insulin [38]. However, GIP is a bifunctional regulator of insulin and glucagon. At hyperglycaemia GIP indeed stimulates insulin release, but in the presence of euglycaemia or hypoglycaemia GIP stimulates glucagon secretion [39]. Plasma glucagon levels are higher during OGTT compared to intravenous isoglycaemic infusion in both healthy individuals and patients with T1D and T2D [27, 37, 40]. Meier et al. [40] showed that the attenuated glucagon inhibition during OGTT was associated with GIP secretion. GIP is most commonly referred to as a product of the K-cells in the intestines, however GIP is also produced in salivary glands [41–43] and stomach [44]. One might speculate that a rapid secretion of GIP in response to glucose intake causes α-cells to release glucagon, accounting for the early peak in plasma glucagon observed in most pigs. However, further research is needed to elucidate the physiological mechanisms behind the shape of the glucagon curve.

During OGTT, plasma levels of active GLP-1 were slightly elevated within 45 min in 4 out of 5 pigs, but compared to humans the increase in active GLP-1 levels was low [28]. Amount of active GLP-1 reaching the circulation after L-cell secretion is however dependent on enzyme activity of dipeptidyl peptidase IV [45] which has not been thoroughly investigated in the pig, and the percentage of secreted active GLP-1 reaching the circulation in pigs is not known. One pig underwent an additional OGTT with a larger glucose load (2.5 g/kg) after which a biphasic GLP-1 curve was seen with peak values at 10 and 60 min after glucose intake, with values 80–90% higher than fasting level. Also in humans active GLP-1 is released in a biphasic pattern with an early phase within 10–15 min and a second phase within 30–60 min. The autonomic nervous system contributes to the rapid release of GLP-1 in the early phase, while the second peak is likely to be caused by direct stimulation of intestinal L-cells by nutrients [38]. As only one pig underwent a 2.5 g/kg OGTT, further experiments are needed to elucidate if the biphasic pattern is true for all pigs receiving this larger glucose load. In the present experiment, an oral glucose load of 1.75 g/kg BW was selected for the young growing pigs as this is the standard dose for children undergoing OGTT. However, the results show that a larger glucose load is more appropriate to closer mimic the human OGTT.

In the present experiment IVGTT was described in young growing pigs. When glucose was infused intravenously, blood glucose levels were rapidly increased and a subsequent fast insulin response was seen. Glucagon was rapidly suppressed, presumably by the high insulin levels. The insulin response lowered blood glucose below fasting values within 30 min. Insulin levels were lowered as blood glucose declined. In response to hypoglycaemia, glucagon was elevated before returning to basal levels. Plasma concentrations of GLP-1 did not change during IVGTT, which was expected since incretin hormones are not released in response to i.v. glucose in other mammals. The pigs were more efficient than humans in eliminating the i.v. glucose load [15, 46], which is in accordance with previous observations in adult pigs by Anderson and Elsley [47].

In conclusion, pigs can be trained to bottle-feed glucose dissolved in water and thereby undergo an OGTT more similar to the human standard OGTT than previously described methods in pigs. The refined method reduces experimental variability, thus facilitates comparison between experiments. Patterns of hormonal secretion in response to oral and intravenous glucose are similar to those in humans; however, pigs are more glucose tolerant with lower insulin levels than humans. This refined porcine OGTT method provides an important new tool for metabolic research in general, and diabetes research in particular.
